# Long-term subjective patient's satisfaction and aesthetic outcomes in sagittal synostosis: a study of 488 patients operated by early, wide, open strip craniectomy

**DOI:** 10.1007/s00381-026-07389-4

**Published:** 2026-07-14

**Authors:** Sapir Sadon, May Brami, Jonathan Roth, David Leshem, Shlomi Constantini

**Affiliations:** 1 Department of Pediatric Neurosurgery, The Pediatric Brain Center, Gilbert Israeli International Neurofibromatosis Center, Dana Children’s Hospital, Tel-Aviv Medical Center, Tel-Aviv University, 6, Weizman, 6423906 Tel-Aviv, Israel; 2 Department of Plastic and Reconstructive Surgery, Tel-Aviv Medical Center, Tel-Aviv University, Tel-Aviv, Israel

**Keywords:** Aesthetic outcomes, Patient satisfaction, Long-term follow-up, Sagittal synostosis, Early surgical intervention

## Abstract

**Objective:**

Infants with sagittal synostosis (SS) are operated upon mainly for cosmetic reasons. The patient's perception related to head shape and to scar, are mostly subjective. We therefore investigated the long term subjective results of relatively homogeneous group of children who were operated on with an early, open, and wide strip craniectomy.

**Methods:**

Single-center, retrospective study with a follow-up of 2 to 25 years. The study cohort included 488 children who underwent early, open, wide, strip craniectomy for SS at the age of 2–4 months and answered the telephone questionnaire. We interviewed the parents, assessing their perception, adding upon the children's related feelings. The questionnaire included both quantitative ratings and qualitative responses "pearls", and assessed aesthetic outcomes, including skull shape, scar-related concerns, and overall satisfaction. The cohort was stratified into three age groups (3–6, 6–12, and ≥ 12 years), and a comparative analysis between males and females.

**Results:**

488 patients responded to the questionnaire. There is an overall very high satisfaction with the surgery's aesthetic results. Except for one patient (1/488), all responders stated that they would undergo the surgery again. Detailed questions about head shape and the influence of the scar, show mostly minor comments that seem to improve when we compare those between 6 and 12, and those above 12 years of age. Scar-related concerns were generally mild in nature (such as local sensitivity, pruritus, or difficulty with haircuts) and did not translate into overall dissatisfaction.

**Conclusions:**

This study confirms that the long-term subjective, aesthetic results of early, wide, open and strip craniectomy of both parents and children are excellent. Despite the presence of minor aesthetic concerns, including scar-related findings, overall satisfaction remains consistently high.

**Supplementary Information:**

The online version contains supplementary material available at 10.1007/s00381-026-07389-4.

## Introduction

The most common form of craniosynostosis is the premature fusion of the sagittal suture (sagittal synostosis, SS), resulting in the characteristic cranial deformity of scaphocephaly or dolichocephaly[1–3]. The primary argument for operating on isolated SS is cosmetic as the condition mainly affects skull shape and appearance [4]. (Jehuda Soleman et al. "Surgery for isolated Sagittal Synostosis: A Neuro-developmental Necessity or an Isolated Cosmetic Challenge? Literature and survey", Child's Nervous System in press) Given the aesthetic argument for the procedure, understanding how patients and their parents perceive surgical outcomes is of utmost importance. Long-term follow-up is particularly crucial, as initial impressions may change over time and subtle concerns about head shape or scar appearance may emerge as the child growth. Several studies have addressed long-term outcomes following surgical correction of sagittal synostosis, consistently demonstrating high levels of satisfaction among patients and parents (Table 1). We conducted detailed interviews with our long-term cohort, focusing on their *subjective* assessment of skull shape and scar, in order to provide a comprehensive evaluation of the long-term aesthetic outcomes of surgical correction for SS. Our study focuses on a highly homogeneous cohort of patients who underwent early surgical intervention (≤ 4 months of age) using a specific technique (open wide strip craniectomy), allowing for a more controlled assessment of outcomes. While surgical outcomes are frequently described as satisfactory, assessing aesthetic success remains complex. Long-term follow-up data of adults treated surgically for sagittal synostosis demonstrate high levels of self-reported satisfaction with appearance [5, 6].

**Table 1:** Review articles

	Article Title **Authors** Year	Journal	Population	Follow-up	Outcome Measured	Method	Results & Main Finding
**1**	Outcomes of 40 Nonsyndromic Sagittal Craniosynostosis Patients as Adults: A Case–Control Study With 26 Years of Postoperative Follow-up Salokorpi 2019^4^	Operative Neurosurgery	40 patients with isolated sagittal synostosis All underwent surgery	Mean follow-up: 26.5 y	Self-reported appearance satisfaction External attractiveness (independent panels)	Patients rated their facial appearance using a 100-mm visual analog scale (VAS)	Excellent long-term outcomes in adulthood, comparable to the general population
**2**	Clinical outcome of the modified pi-plasty procedure for sagittal synostosis Guimarães-Ferreira 2001^7^	Journal of Craniofacial Surgery	110 patients with non-syndromic single-suture sagittal synostosis	Evaluation at 3 and 5 y	Parental satisfaction questionnaire	Cephalometric radiographs and questionnaire	High level of satisfaction was found among the parents with the aesthetic result
**3**	Nonendoscopic, minimally invasive calvarial vault remodeling without postoperative helmeting for sagittal synostosis Mutchnick 2012^8^	Journal of Neurosurgery Pediatrics	18 infants with Sagittal synostosis	Mean 16.4 m	Morphological index and parental questionnaire	Retrospective cohort; nonendoscopic minimally invasive CVR (strip craniectomy with barrel-stave osteotomies) with CI measurement and parental questionnaire follow-up	92% were happy that they were spared the need to use a helmet
4	Craniofacial characteristics and cosmetic satisfaction of patients with sagittal and metopic synostosis: a case–control study using 3D photogrammetric imaging Svalina 2022^5^	Child's Nervous System	49 adult patients above 18 years Treated for sagittal and metopic	Very Long-term	Objective symmetry measures External aesthetic assessment Subjective satisfaction	Objective symmetry analysis (landmark-based & mirror-shell); expert and layperson appearance ratings (VAS 0–100); patient self-assessment; cephalic index (multi-modal measurement); clinical head shape evaluation; perioperative data (operative time, blood loss, LOS); pre-op imaging ± selective post-op	Gap between scientific measurements and the patient's subjective feeling
5	Quality of life and satisfaction in surgical versus conservative treatment of nonsyndromic children with craniosynostosis Sader 2021^9^	Journal of Neurosurgery Pediatrics	114 children with single-suture craniosynostosis 78% operated (< 24 months)(54% sagittal suture)	Median age 3.5 Y (2–6 Y)	Quality of life Satisfaction Reasons for choosing treatment	PedsQL (Pediatric Quality of Life Inventory) questionnaire	Parents chose surgery because of "severe appearance" of the head
6	Child-Patient Perspective on Results After Correction of Sagittal Synostosis and the Difference Between Child-Patient and Parent's Perspectives Kurniawan 2024^10^	Journal of Craniofacial Surgery	96 operated patients operated for sagittal synostosis	6 to 18 y	- Patient satisfaction: Self-assessment of the results of the surgery	Questionnaires (patients and their parents)	Agreement between parents and children was only 49.7% on average
7	Long-term parental satisfaction and cosmetic, ophthalmological, and cognitive outcomes after sagittal strip craniectomy with barrel stave Pinar 2025^**11**^	Journal of Neurosurgery, Pediatrics	25 patients operated for sagittal synostosis	Minimum follow-up period for patients was 3 years	Primary outcome: Parental satisfaction reported in questionnaires	Use of Parental Self-Report Questionnaires (PROs) to measure satisfaction	High satisfaction: 96% of parents, 94.7% of surgeons; 89.5% parent–surgeon aesthetic agreement. CI normal in 78%
**8**	Patient-reported outcome measures more than fifteen years after treatment of sagittal or metopic craniosynostosis: a prospective cohort study Woerdeman 2024^12^	Child's Nervous System	A total of 36 respondents (out of 52 consenting patients from an initial cohort of 253), predominantly with sagittal craniosynostosis (29/36), including 29 operated and 7 non-operated patients, all of whom completed the CSO-Q questionnaire	Long-term follow-up of at least 15 years, with a mean of approximately 20 years	Anthropometric cranial measurements and patient-reported outcomes including cosmetic satisfaction (CSO-Q), self-esteem (RSES), and fear of negative evaluation (FNE)	Prospective cohort study comparing operated and non-operated patients using questionnaires and clinical physical measurements	Cosmetic satisfaction and psychological outcomes were similar between operated and non-operated groups, with overall high satisfaction rates
**9**	Surgical Technique and Validation of Outcome Assessment in Sagittal Synostosis Psaras 2010^13^	Central European Neurosurgery	87 children who underwent surgical correction for ss	Aesthetic outcomes were assessed at 6, 12, and 18 months postoperatively	Aesthetic outcomes evaluated using a 5-grades	Retrospective review surgical technique involved bilateral total removal of the parietal bones, allowing bone regeneration guided by the expanding brain	Most patients (79.3%) achieved excellent or good aesthetic outcomes as rated by both parents and physicians
**10**	Review Article- Cosmetic satisfaction and patient-reported outcomes following surgical treatment of single-suture craniosynostosis: a systematic review Klieverik 2023^**14**^	Child's Nervous System	724 patients (data from 12 studies)		Patient and family perception of aesthetic and functional results; cosmetic satisfaction	Literature review of 12 studies; measurements included VAS, yes/no questions, and 5-point scales	Overall improvement in cosmetic satisfaction post-surgery

However, the literature lacks several importent sub-analysis of parents and children satisfaction, E.g. the differences between male and female patients, and stratification by age. In particular, school-age children are not commonly analyzed as a distinct population, despite the potential influence of age-related differences in social interaction, perception, and self-awareness on subjective outcomes.

The present study aimed to evaluate aesthetic outcomes following early surgical correction of sagittal synostosis, emphasizing the combined *subjective* impressions of both patients and parents, with particular attention to school-age children.

## Methods

The study was conducted as a retrospective, single-center analysis and was approved by the hospital's research committee (IRB). Telephone consent was obtained from the participants. We administered a telephone questionnaire to parents of patients who underwent early open wide strip craniectomy for sagittal craniosynostosis, in order to evaluate long-term personal, social, and aesthetic outcomes. Inclusion criteria comprised patients with isolated sagittal synostosis who underwent early surgical repair between 1999 and 2022, at an average age of 3.2 months, and who completed the telephone questionnaire. Patients were stratified into three age groups (3–6, 6–12, and ≥ 12 years). For children aged ≥ 12 years, both parental and child-reported responses were included.

Along the years we performed the same surgical procedure for infants at an early stage with SS. In short, babyies are posioned prone on a been bag. The scar is curvy and inlonding from behind the hairline to the inion. The surgical bone is removed widly 60–70 mm side bone cuts with greenstick fractures and lateral benging allowing further immediate widening. Skin was closed with absorbable sutures. The avarage time is 35–40 min. The length of hospitalization is 3–4 days.

The questionnaire (Appendix A) was structured into several domains, including:

- Surgical results
- Social/personal
- Follow-ups
- Reflective
- General questionnaire

The questionnaire included both quantitative ratings and qualitative responses (“pearls”), providing additional insight into the subjective experiences of both patients and their parents. (Appendix B).

Statistical analysis was conducted using simple t-tests and chi tests.

## Results: (see Table 2)

**Table 2:** Overview of the results

Population	Total patients identified	507
	Included in study	488 (96.3%)
	Non-responders	3.7% (2 declined, others unreachable/no updated contact details)
	Male sex	397
Timing	Mean age at surgery	3.2 months
	Mean age at questionnaire	8.1 years (range 2–25)
Age groups	2–6 years	*n = *142
	6–12 years	*n = *190
	≥ 12 years	*n = *156
Parent reasonfor surgery	Aesthetic reasons	93.7%
	Concern for elevated ICP	2.7%
	Developmental concerns	4.8%
Satisfaction (Parental response)	Parents satisfied child underwent surgery	99.79%
	Parents are satisfied with the result	95.2%
	Child satisfaction	92.6%
	Satisfaction (6–12 years)	95.3%
	Satisfaction (≥ 12 years)	97.4%
	Would not choose surgery again	1 patient
Aesthetic score (0–100)	Overall	87.6 ± 13.3
	6–12 years	86.4 ± 14.8
	≥ 12 years	87.0 ± 13.7
General concerns about the appearance of the head	6–12 years	15.3%
	≥ 12 years	8.3%
Scar-related issues	Haircut difficulty (6–12/≥ 12)	7.9%/3.8%
	Pruritus (6–12/≥ 12)	1.6%/0.6%
	Local sensitivity (6–12/≥ 12)	1.0%/0.6%
Head shape concerns	6–12 years	~ 5%
	≥ 12 years	~ 4%
Social impact	Teasing (6–12/≥ 12)	6.3%/3.2%
	Direct bullying	0.6% (1 patient, ≥ 12 group)
Clinical outcomes	Postoperative ICP	3 patients (all resolved)
	Secondary cosmetic surgery	1 patient (occipital hump)
	Mortality	0
	Significant morbidity	None

This retrospective review initially identified 507 patients diagnosed with early SS who underwent surgery between 1999–2022 at an average age of 3.2 months. Of those, 488 (96.3%) patients (397 male) responded to the detailed telephone questionnaire at a mean age of 8.1 years (range, 2–25 years) about their experience during and after the surgery, and were included in this study. The remaining 3.7% consisted of 2 patients who declined participation, while the others could not be reached or had no updated contact information available. For further analysis, patients were divided into three age groups: Children aged 2–6 years (*n = *142), children aged 6–12 years (*n = *190), and children aged ≥ 12 years (*n = *156). We collected demographic data, gender, age, family history, complications during surgery, length of hospitalization, comorbidity, mortality, follow-up after surgery, and cosmetic results.

Alongside the quantitative data, qualitative expressions (“pearls”) were also collected, providing additional insight into the patients’ and their parents’ *subjective* experience.

### Why did you decide to do the surgery?

The most commonly reported reasons for deciding to undergo surgery were expectations of achieving a better final aesthetic result, as well as concerns regarding potential neurological damage and elevated intracranial pressure (ICP). These themes consistently emerged as the primary factors influencing parental decision-making. Notably, the vast majority of respondents (93.7%, 389/415) indicated that aesthetic considerations were the primary driver for surgery, whereas only a small proportion cited concerns regarding elevated intracranial pressure (2.7%, 11/415) or potential developmental issues (4.8%, 20/415).

### Parent satisfaction following surgery

Parental satisfaction following surgery was very high. Overall, 99.79% of parents reported that they were happy that their child had undergone the procedure. Only one respondent stated, in retrospect, that they would not have chosen to undergo the surgery. 95.2% of parents expressed satisfaction with the surgical result, while 92.6% reported that their child was satisfied with the outcome. Child satisfaction was assessed based on parent-reported responses obtained after consultation with the child, as described in the Methods section. Even among the less satisfied respondents, the surgical results were commonly described as “good” or “excellent.” Among children aged 6–12 years (*n = *190), 181 (95.3%) were satisfied, while 9 (4.7%) were dissatisfied. In the older age group (≥ 12 years; *n = *156), 152 children (97.4%) expressed satisfaction, and 4 (2.6%) reported dissatisfaction. These findings indicate that the small number who expressed dissatisfaction at ages 6–12 became more satisfied at ages ≥ 12, with consistently high levels of satisfaction across both age groups.

### How would you rate the aesthetic result on a scale between 0–100?

**Fig. 1:**
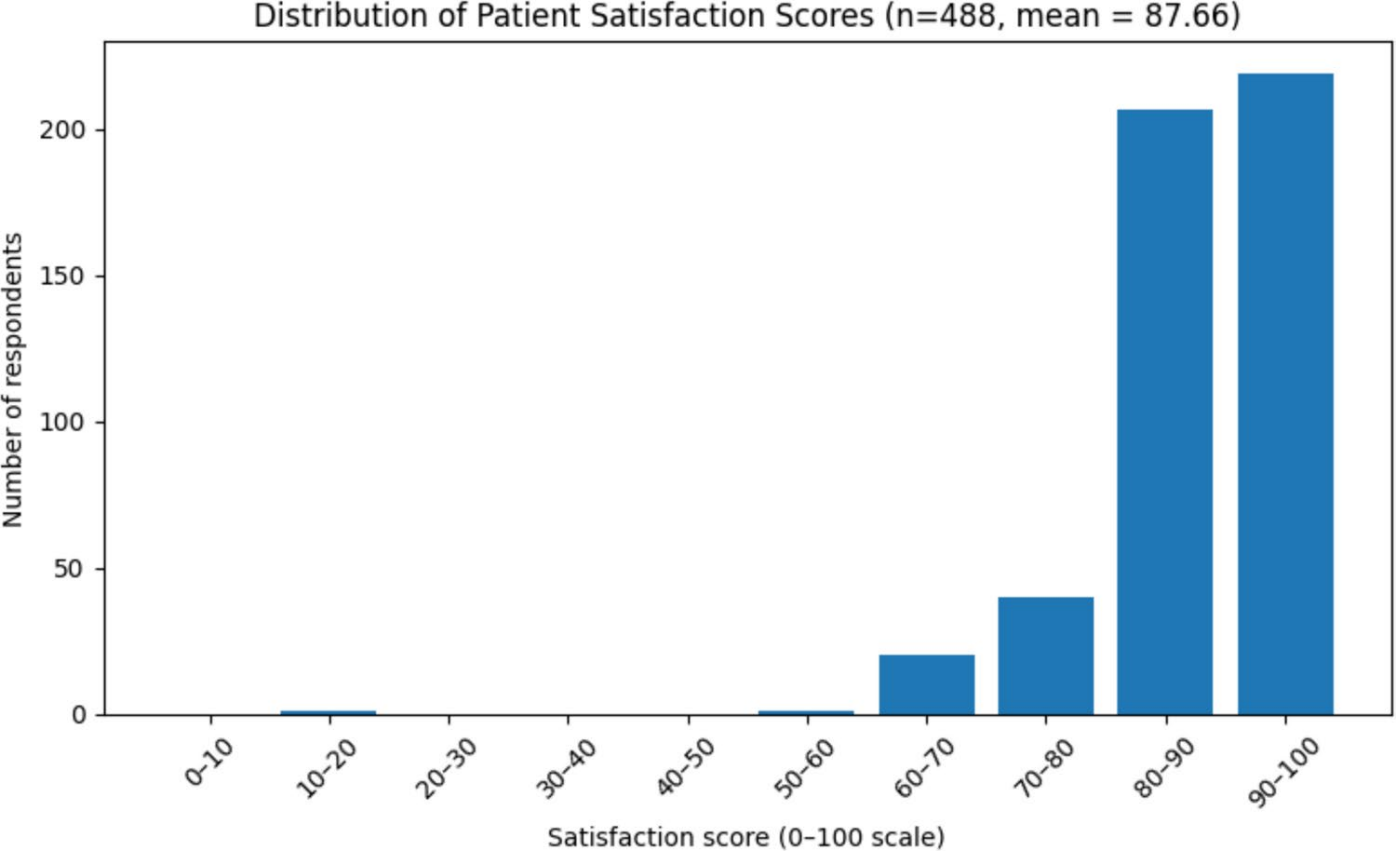
Distribution of aesthetic scores among patients

The overall mean score was 87.6 ± 13.3 (*n = *488). For the assessment of children’s own opinions, only data from children aged ≥ 6 years was included, as these are ages at which children are able to reliably express their views. It is important to note that the parents answered the question, but were asked to consult their child when providing the response. Among children aged 6–12 years, the mean score was 86.4 ± 14.8 (*n = *190), with a mean score of 85.8 for females and 86.7 for males, while in those aged ≥ 12 years, the mean score was 87.0 ± 13.7 (*n = *156). with a mean score of 86.7 for males and 88.6 for females. No statistically significant differences in aesthetic scores were observed between males and females in either age group (6–12 years: *p* = 0.76; ≥ 12 years: *p* = 0.31, independent t-test). These findings are illustrated in Fig. 1.

The following paragraphs are based on parent-reported responses, with parents instructed to obtain their child’s input when answering.

### Is there something about the head that bothers you?

Parents were asked whether there was any aspect of their child’s head that was bothersome after consulting with their child. Among children aged 6–12 years (*n = *190), 29 (15.3%) reported concerns related to the scar or head shape. Of these, 16 of 150 boys (10.7%) and 2 of 40 girls (5.0%) specifically reported that the scar was bothersome. In the older age group (≥ 12 years; *n = *156), 13 children (8.3%) expressed similar concerns, including 7 of 105 boys (6.7%) and 4 of 46 girls (8.7%) reporting scar-related complaints. Overall, only a minority of children reported dissatisfaction with specific aspects of head appearance. Details of the specific concerns are provided below.

### Patient satisfaction vs. specific soncerns

In both age groups, more children reported specific aesthetic concerns than expressed overall dissatisfaction with the surgical outcome. This indicates that minor issues, such as scar appearance or subtle head shape differences, do not necessarily affect overall satisfaction with the surgery.

### Scar related issues

The majority of children reported minimal problems. Among children aged 6–12 years (*n = *190), 15 (7.9%) reported difficulty with haircuts due to the scar, 3 (1.6%) reported pruritus, and 2 (1.0%) reported local sensitivity. In the older age group (≥ 12 years; *n = *156), 6 children (3.8%) reported difficulty with haircuts, 1 (0.6%) reported pruritus, and 1 (0.6%) reported local sensitivity. Overall, the scar was not bothersome for the vast majority of children, and most comments regarding the scar were minor in nature (Table 3). Among children aged 6–12, 16 out of 150 boys (10.7%) and 2 out of 40 girls (5.0%) reported that the scar was bothersome. In the 12 + age group, 7 out of 105 boys (6.7%) and 4 out of 46 girls (8.7%) reported bothersome scars. Overall, scar-related complaints were low in both age groups. No significant association was found between gender and scar-related complaints in either the 6–12 age group (p = 0.25) or the 12 + age group (*p* = 0.65). While a slightly higher proportion of boys reported complaints in the younger group (10.7% vs. 5%), proportions were similar in the older group (6.7% vs. 8.7%).

### Head shape concerns

A small proportion of children reported concerns regarding the shape of their head. Among children aged 6–12 years (*n = *190), 4 (2.1%) reported small wave-like ridges or asymmetry, 1 (0.5%) reported a protrusion at the occipital region, 1 (0.5%) noted a prominent bony area at the crown, and 2 (1.0%) reported other or general head shape concerns. Of these, 6 of 150 boys (4.0%) and 2 of 40 girls (5.0%) specifically reported that the head shape was bothersome. In the older age group (≥ 12 years; *n = *156), 2 children (1.3%) reported small wave-like ridges or asymmetry, 2 (1.3%) reported a protrusion at the occipital region, and 2 (1.3%) reported other or general head shape concerns, including 4 of 105 boys (3.8%) and 2 of 46 girls (4.3%) who specifically reported concerns. Overall, the head shape was not bothersome for the vast majority of children, and most comments were minor in nature. (Table 3), with only one patient undergoing secondary surgery for cosmetic concerns related to head shape. No significant association was found between gender and head shape complaints in either age group (*p* = 0.9).

Additionally, three patients developed elevated intracranial pressure (ICP) later postoperatively, all of whom were successfully managed (two with cranial vault expansion and one with acetazolamide), with complete resolution of papilledema and no lasting neurological sequelae. Only one child was operated for cosmetic reasons, occipital hump. No mortality or significant morbidity was observed [7].

The overall neurodevelopmental information was described in a separate pepper who is part of ISPN, child nervous system collection (The incidence of subsequent high intracranial pressure in patients undergoing early, open, and wide strip craniectomy for sagittal synostosis) [7].

In short only, 20/488 patients reported an active neurological diagnosis. Reported diagnoses included learning disability (11 patients), speech delay (3 patients), developmental delay (1 patient), seizures (2 patients), cerebral palsy (1 patient), FOXP1 syndrome (1 patient), and autism spectrum disorder (1 patient). Overall, less than 1% of the patients underwent an additional procedure following the initial surgery. These results were reported in a separate paper.

### Social environment

It was important to assess how the child’s social environment responded to their appearance following surgery. Among children aged 6–12 years (*n = *190), 12 (6.3%) reported teasing related to the scar. No cases of direct ridicule or bullying were reported; all instances involved repeated questions from friends or adults, which were perceived as teasing by the children. In the older age group (≥ 12 years; *n = *156), 5 children (3.2%) reported teasing, including 1 case of direct ridicule or bullying (0.6%) and 4 cases (2.6%) involving repeated questions from friends or adults perceived as teasing. Overall, reports of social challenges related to appearance were very uncommon in both age groups. (See Appendix B).

## Discussion

This study focused on the *subjective* experience of patients and parents following surgical correction of isolated sagittal synostosis (SS). This is a large, homogeneous cohort that underwent early, wide, open strip craniectomy. Therefore, the following results are relevant to this group only, and the emphasis was placed on long-term satisfaction reported by both patients and their caregivers. In a different pepar we reframed to the neurodevelopmental aspects of the same group(Jehuda Soleman et al. "Surgery for isolated Sagittal Synostosis: A Neuro-developmental Necessity or an Isolated Cosmetic Challenge? Literature and survey", Child's Nervous System in press). Being a survey-based study, we collected not only numerical data but also textual responses (“pearls”), which are highly relevant to patients’ and parents’ feelings and provide meaningful personal insights (Appendix B). This is also the largest study looking, in some depth, into the long-term effects of the scar. We also stratified our results by age groups.

The present findings demonstrate consistently high levels of long-term satisfaction among both patients and parents following surgical correction of isolated sagittal synostosis (SS), broadly consistent with existing literature. Reported studies in the literature affirm good long term results, however they vary in surgical technique, age at surgery (early/late), follow up durations and assessment methods (See Table 1).

The present study focuses exclusively on SS that operated openly and early (around 3 months). It focuses exclusively on *isolated* SS, allowing for a more homogeneous study population. All patients were treated using an identical surgical technique, enabling consistent evaluation of outcomes without variability related to different operative approaches and ages. We managed to achieve a very high response rate to the questionnaire, strengthening the validity and the reliability of the findings. Finally, the cohort was stratified by gender and into distinct age groups, with particular emphasis on school-age children, allowing for age-specific analysis of subjective outcomes during a critical developmental period.

The results demonstrate consistently high levels of long-term satisfaction among both parents and patients. The perception of positive surgical outcomes was there even when minor aesthetic concerns are present. Interestingly, while some children reported specific concerns regarding the scar or head shape, these concerns did not translate into overall dissatisfaction with the surgical outcome. It appears that with increasing age, the perceived impact of aesthetic concerns diminishes, as older children reported fewer disturbances compared to younger ones. The social impact of these findings was extremely limited. Some reported questions and perhaps mild teasing related with interactions such as repeated questions or mild teasing, not leading to a significant negative effect on the patients’ overall experience. Taken together, these findings suggest that minor aesthetic imperfections do not play a central role in determining overall satisfaction with the surgery (Appendix B).

In a case–control study, Salokorpi et al. evaluated long-term outcomes of 40 patients with nonsyndromic sagittal craniosynostosis who underwent surgery in childhood, at a mean age of 5.7 months, compared with 40 age- and gender-matched controls, after a mean follow-up of 26.5 years. Aesthetic outcomes were assessed using standardized facial photographs rated by two independent panels (dentists and laypersons) using a visual analog scale (VAS), and participants also completed self-reported questionnaires regarding satisfaction with appearance, socioeconomic status, and general health. Patients reported similar satisfaction with their facial appearance compared with controls, although external panel evaluation rated patients as slightly less attractive. No significant differences were found between groups in general health, mental health, education level, employment status, or family situation. Study limitations include the relatively small patient cohort and lack of data regarding the preoperative severity of the deformity. Given that these patients underwent surgery at a later age, this may reflect the use of different surgical techniques and could be associated with comparatively less favorable outcomes [5].

In a retrospective study, Guimaraes-Ferreira et al. evaluated the outcomes of the modified pi-plasty technique for the treatment of nonsyndromic sagittal craniosynostosis in 110 patients (mean age at surgery: 7.73 months). Outcomes were assessed using cephalometric radiographs obtained preoperatively and postoperatively at ages 3 and 5 years, as well as parental questionnaires evaluating aesthetic outcomes. The procedure resulted in significant improvements in skull morphology, with the cephalic index increasing from 65% preoperatively to 72% postoperatively, and parents reporting significant improvement in overall skull appearance. The procedure demonstrated low morbidity and no mortality, supporting its safety. The main limitation of the study is the relatively short follow-up, limited to age 5, which does not allow assessment of long-term outcome. Additionally, although parental questionnaires were used, the specific questionnaire items were not fully detailed.The relatively older age at surgery may also indicate the use of different surgical approaches, which could impact outcomes and potentially be associated with less favorable results compared to earlier intervention [8].

In a retrospective single-institution cohort study, Mutchnick and Maugans evaluated a minimally invasive calvarial vault remodeling technique performed without endoscopic assistance and without postoperative helmet therapy for the treatment of nonsyndromic sagittal craniosynostosis in 18 infants (mean age at surgery 2.3 months). Outcomes assessed included operative variables, complications, cephalic index (CI), and caregiver-reported cosmetic satisfaction using a 5-item nonstandardized questionnaire administered to caregivers. After a mean follow-up of 16.4 months, a significant improvement in CI was observed, increasing from 69 preoperatively to 79 postoperatively. No mortality was reported and only one superficial postoperative infection occurred. Most caregivers reported satisfaction with the cosmetic outcome and appreciation for avoiding postoperative helmet therapy. Limitations of the study include the very small patient cohort and relatively very short follow-up, which limit the ability to assess long-term outcomes [9].

In a prospective case–control study, Svalina et al. evaluated craniofacial characteristics and aesthetic outcomes in patients who underwent surgery in early childhood for nonsyndromic sagittal or metopic craniosynostosis. The exact age at surgery was not specified in the study. The study included 49 patients (41 sagittal and 8 metopic) and 65 age- and gender-matched controls. Only patients who were 18 years or older at the time of evaluation were included, reflecting long-term follow-up into adulthood after surgery in early childhood. Facial morphology and symmetry were assessed using 3D photogrammetric imaging with landmark-based analysis, and satisfaction with appearance was evaluated using questionnaires and panel assessment of photographs using a VAS scale. However, the content and structure of the questionnaire were not detailed. Patients demonstrated slightly greater facial asymmetry, particularly in the forehead region, and a greater intercanthal distance compared with controls; however, the differences were small (< 3 mm) and not clinically significant. Limitations of the study include the *subjective* nature of aesthetic evaluation and the focus on soft-tissue facial characteristics rather than underlying skull morphology. In addition, the lack of detailed reporting regarding age at surgery and specific diagnostic subgroup analyses (e.g., by synostosis type) may limit the ability to fully interpret and generalize the findings [6].

In a multicenter cross-sectional study, Sader et al. evaluated quality of life and parental satisfaction among children with nonsyndromic single-suture craniosynostosis managed either surgically or conservatively. The study included 114 children, of whom 78% underwent surgery and 22% received conservative management. Quality of life was assessed using the Pediatric Quality of Life Inventory (PedsQL), and parental satisfaction was evaluated using Likert scales. Among those who underwent surgery, the median age at surgery was 4 months. The median age at survey was 3.5 years, reflecting relatively short-term follow-up. On univariate analysis, higher quality-of-life scores were observed in the conservatively managed group, but after adjusting for deformity severity and age at diagnosis, no significant association between treatment type and quality of life was found. Parental satisfaction was high and similar between groups. The main limitation of the study is the short follow-up period. In addition, although validated questionnaires were used, the specific questionnaire items were not detailed [10].

In a questionnaire-based study, Kurniawan et al. evaluated patient satisfaction after surgical correction of sagittal synostosis and compared the perspectives of children and their parents. The questionnaire was completed by 96 patients aged 6–18 years, with a median age of 11.6 years at evaluation, reflecting long-term follow-up after surgery. According to the surgical protocol, extended strip craniotomy (ESC) was performed in children presenting before the age of 6 months, whereas frontobiparietal remodeling (FBR) was performed in those presenting after 6 months of age; however, the exact age at surgery for the cohort was not reported. Patients had undergone either frontobiparietal remodeling or extended strip craniotomy. Most patients (81.2%) reported that their head shape was similar or only slightly different from others, and 60% indicated that the scar was barely noticeable. However, 33% reported that they would change some aspect of their head shape, and 29.1% reported experiencing headaches. Agreement between child and parent responses was 49.7% on average. A limitation of the study is the relatively small patient cohort. Limitations of the study include the relatively small patient cohort and the use of mixed surgical techniques within the study population, which may confound the interpretation of outcomes [11].

In a single-center study, Pinar et al. evaluated parental satisfaction and cosmetic, cognitive, ophthalmological, and quality-of-life outcomes after surgical treatment of nonsyndromic sagittal craniosynostosis. The study included 25 patients who underwent surgery before the age of 1 year using midline strip craniectomy with barrel stave osteotomies and occipital release, with a minimum follow-up of 3 years. Ninety-six percent of parents reported satisfaction with the surgical outcome, and the children demonstrated age-appropriate development and high quality of life. Limitations of the study include the small patient cohort and relatively short follow-up [12].

Woerdeman et al. reported long-term outcomes following surgical and non-surgical management of sagittal and metopic craniosynostosis, with a mean follow-up of approximately 20 years. Within the sagittal group, 39 patients were included, of whom 34 underwent surgery at a median age of approximately 5 months, and only 29 participated and completed the questionnaire. The surgical procedures included various open cranial vault techniques based on vertex craniotomy. Overall cosmetic satisfaction was high (~ 80%) regardless of treatment status. However, the study is limited by a small sample size, low participation rate, heterogeneity of surgical techniques, potential selection bias, and the relatively late age at surgery [13].

Psaras et al. analyzed 87 children with sagittal synostosis who underwent surgical correction using an osteoclastic technique involving bilateral parietal bone removal, allowing spontaneous skull remodeling. The median age at surgery was 5 months, and outcomes were assessed at 6, 12, and 18 months. Good to excellent aesthetic results were reported in 79.3% of cases, with low rates of significant asymmetry (5.7%) and reoperation (1.1%), alongside a high concordance between parental and medical team evaluations. The authors suggest that the optimal timing for surgery is 5–6 months of age. Study limitations include a relatively short follow-up and the relatively late age at surgery [14].

In a systematic review, Klieverik et al. evaluated cosmetic satisfaction and patient-reported outcomes following surgical treatment of single-suture craniosynostosis. The review included 12 studies comprising 724 patients, in which outcomes were assessed using questionnaires and interviews. Overall, the studies demonstrated a trend toward improved aesthetic appearance after surgery, although no clear overall trend in patient-reported outcomes was identified. A major limitation of the review is the substantial heterogeneity in outcome measures across the included studies, which prevented direct comparison between them [15].

We managed to achieve a very high response rate to the questionnaire, strengthening the validity of the findings. Finally, the cohort was stratified into gender and distinct specific age groups, with particular emphasis on school-age children, allowing for age-specific analysis of subjective outcomes during a critical developmental period.

This study may help inform clinical decision-making by providing long-term patient- and parent-reported outcomes following early, open surgical correction of isolated sagittal synostosis. The consistently high levels of satisfaction, minimal social impact, and low rates of secondary intervention support the role of early surgical intervention when aesthetic concerns are the primary indication for treatment. This data may also contribute to shared decision-making by helping parents better understand the balance between potential minor residual aesthetic findings and overall long-term satisfaction.

To note that these findings are relevant for a specific technique and age group, alternative surgical methodologies should also look into the long term results in a similar fashion.

## Conclusions

The present study demonstrates that early surgical correction of isolated sagittal synostosis using an open, wide strip craniectomy is associated with consistently high long-term satisfaction among both patients and their parents. Despite the presence of minor aesthetic concerns, particularly related to the scar or subtle variations in head shape, these findings rarely translate into overall dissatisfaction. Importantly, the perceived impact of such concerns appears to diminish with age, suggesting an adaptive component over time. The minimal social impact and the low rate of secondary interventions further support the favorable long-term outcomes of this approach.

Given the homogeneous cohort, standardized surgical technique, and high response rate, these findings provide robust insight into subjective aesthetic outcomes following early intervention. However, the results should be interpreted in the context of this specific surgical approach and patient population.

## Limitations

This study has several limitations that should be acknowledged. First, the assessment of outcomes was based on *subjective* reports, without the use of objective cranial measurements such as the cephalic index (CI). In addition, responses were provided by a single parent (either mother or father) for each patient. These findings reflect the outcomes of a specific surgical technique and age group, and similar long-term evaluations of alternative surgical approaches are warranted. Finally, the study population included diverse subgroups (e.g., secular Jewish, ultra-Orthodox, Arab, and immigrant populations), which may differ substantially in cultural perceptions, expectations, and reporting of outcomes.

## Electronic supplementary material

Below is the link to the electronic supplementary material.

Supplementary file1 (DOCX 22 KB)

## Data Availability

No datasets were generated or analysed during the current study.

## Appendix B: Selected patient quotations are presented below, organized by age and thematic relevance

**Table 3:** Selected patient quotations ("pearls")

Scar related issues:	
Children aged 6–12 Y (*n = *190):	Children aged above 12 Y (*n = *156):
"The scar came out wide." "The scar is aesthetically bothersome to the child; when he gets a haircut and it is visible, children ask about it." "During seasonal transitions, the scar itches and bothers him during haircuts." "Touch sensitivity in the scar area." "The scar is aesthetically bothersome, wide, prominent, and does not allow a short haircut." "The child cannot get a very short haircut." "Aesthetically, the scar is bothersome, wide, prominent, and does not allow a short haircut. The head shape itself is completely normal."	"The scar and head shape bother her." "The scar bothers her aesthetically—it is wide, prominent, and prevents her from having short hair." "The scar bothers her aesthetically." "The scar bothers her."
Head Shape:	
Children aged 6–12 Y (*n = *190):	Children aged above 12 Y (*n = *156):
"The skull is asymmetrical; the hair grows in different directions, creating a haircut problem." "The shape of the head (bumps) bothers the child." "Approximately 10 cm above the nape there is a prominent bone; the scar is aesthetically bothersome." "The scar and the shape of the head bother her." "The skull did not close and is not smooth; the scar is visible." "The scar and the shape of the head bother the child."	“Her skull is small and more triangular.” “There is a bulge on the skull.” “There is a problem with the head shape; there is a bulge.” “The skull is asymmetric; hair grows in different directions, creating a problem with hairstyle.”
Social challenges:	
Children aged 6–12 Y (*n = *190):	Children aged above 12 Y (*n = *156):
"children ask about it." "Several teachers asked him about it, made him uncomfortable." "Whenever an adult asked about the scar, the child would shrink with discomfort." "It was not exactly teasing, but rather repeated questions."	"One day he came home from school, I think he was in first or second grade, and I noticed something was wrong. After some questioning, I understood something had happened on the bus. Eventually, the child said that children on the bus had laughed at him and humiliated him because of his different appearance." "He receives many questions about the scar; when he was younger, he used to lie about it."
