# Supplementary material for: Long-term subjective patient's satisfaction and aesthetic outcomes in sagittal synostosis: a study of 488 patients operated by early, wide, open strip craniectomy
Source: Childs Nerv Syst. 2026 Jul 14;42(1):294. doi: 10.1007/s00381-026-07389-4 (PMC13369719; doi:10.1007/s00381-026-07389-4)
Supplement: Supplementary file 1 — Supplementary file1 (DOCX 22 KB) [file 381_2026_7389_MOESM1_ESM.docx]

**Appendix A:**

First Questionnaire:

**Parent Questionnaire (Translated from Hebrew)**

Do the parents agree to answer the questionnaire? Yes / No

**A. Technical / Demographics:**

1. Date of questionnaire completion:
2. Patient’s age at the time of the questionnaire:
3. Who is answering the questionnaire? Father / Mother / Other:
4. How many siblings does the child have and what is his/her position in the birth order?

**B. Results:**

1. Are you (the parent) satisfied with the result? Yes / No / comment:
2. Are you satisfied that your child underwent the surgery? Yes / No / comment:
3. Is the child satisfied with the operative result? Yes / No / comment:
4. How would you rate the aesthetic result on a scale between 0–100?
5. If you had to describe it in words: Poor / Fair / Good / Very good / Excellent

**C. Social / Personal:**

1. Has your child experienced teasing as a result of the appearance after the surgery? Yes / No
2. Was / is there currently something that bothers him/her as a result of the process? Yes / No
3. Is there something about the head that bothers him/her now? Yes / No
4. Does the scar bother him/her? Yes / No
5. Comments:

**D. Follow-up:**

1. Has your child undergone any further procedure following the surgical operation? Yes / No
2. Which procedure did he/she undergo? (if you answered yes to the previous question)
3. Is your child currently under neurological follow-up? Yes / No
4. What is the neurological diagnosis? (if any)
5. Does your child have neurological difficulties? Yes / No
6. If you answered yes to the previous question, what are the difficulties?
7. Is your child under ophthalmologic (eye) follow-up? Yes / No
8. If so, what is the diagnosis?

**E. Reflection:**

1. Looking back – would you undergo the surgery? Yes / No
2. Why? *text*
3. What was the most difficult part of the process for you?
4. In your opinion, is there anything that can be done to make the process easier?

**F. Other:**

1. Did the child undergo evaluation at the army recruitment office? Yes / No
2. What is his medical army profile? (max is 97)
3. If the profile is not 97, what was it lowered for?
4. Did he enlist? Yes / No
5. If not, why? (if you answered no to the previous question)
6. Gender:
7. Household monthly income level relative to the average?
   Low / Average / High / Refuses
   (Current average monthly income in Israel is approximately 13,000 NIS)
8. If there is anything else you would like to share with us, this is the place –

**Thank you for your cooperation!**

Second Questionnaire:

**Supplementary Questionnaire (for 133 patients who reported neurological follow-up at the time of the previous questionnaire) (Translated from Hebrew)**

1. Does your child attend a regular school? (Yes/No):
   comments if applicable:
2. Does your child have difficulty in any school subject, for example mathematics? (Yes/No): please specify:
3. Is/was there a personal aide in kindergarten/school? (Yes/No):
   comments if applicable:
4. Has your child undergone genetic testing? If yes, what were the results? (Yes/No): if yes, please specify the results:
